# Decorating phenylalanine side-chains with triple labeled ^13^C/^19^F/^2^H isotope patterns

**DOI:** 10.1007/s10858-024-00440-z

**Published:** 2024-03-21

**Authors:** Giorgia Toscano, Julian Holzinger, Benjamin Nagl, Georg Kontaxis, Hanspeter Kählig, Robert Konrat, Roman J. Lichtenecker

**Affiliations:** 1 Christian Doppler Laboratory for High-Content Structural Biology and Biotechnology, Institute of Organic Chemistry, University of Vienna, Währinger Str. 38, 1090 Vienna, Austria; 2 Christian Doppler Laboratory for High-Content Structural Biology and Biotechnology, Department of Structural and Computational Biology, Max Perutz Labs, University of Vienna, Campus Vienna Biocenter 5, 1030 Vienna, Austria; 3 Vienna Doctoral School in Chemistry (DoSChem), University of Vienna, Währinger Str. 42, 1090 Vienna, Austria; 4 Institute of Organic Chemistry, University of Vienna, Währinger Str. 38, 1090 Vienna, Austria; 5 Department of Structural and Computational Biology, Max Perutz Labs, University of Vienna, Campus Vienna Biocenter 5, 1030 Vienna, Austria; 6 MAG-LAB, Karl-Farkas-Gasse 22, 1030 Vienna, Austria

**Keywords:** Fluorine NMR, Fluorophenylalanine, Isotope labeling, F-TROSY, Protein overexpression

## Abstract

We present an economic and straightforward method to introduce ^13^C-^19^F spin systems into the deuterated aromatic side chains of phenylalanine as reporters for various protein NMR applications. The method is based on the synthesis of [4-^13^C, 2,3,5,6-^2^H_4_] 4-fluorophenylalanine from the commercially available isotope sources [2-^13^C] acetone and deuterium oxide. This compound is readily metabolized by standard *Escherichia coli* overexpression in a glyphosate-containing minimal medium, which results in high incorporation rates in the corresponding target proteins.

**Supplementary Information:**

The online version contains supplementary material available at 10.1007/s10858-024-00440-z.

## Introduction

Fluorine-19 is constantly gaining importance as a sensitive reporter in the NMR study of large biomolecules. This NMR sensitivity results from several beneficial properties of this nucleus, such as a nuclear spin of 1/2, 100% natural abundance, and a favorable gyromagnetic ratio. Therefore, fluorine is the only element whose NMR signal sensitivity comes close to that of the hydrogen nucleus (~ 83% relative to ^1^H). The ^19^F-NMR signal covers a wide chemical shift range (~ 300 ppm) and responds with major signal shift perturbations to alterations in the electron environment, as the fluorine lone-pair electrons are highly sensitive to changes in their chemical environment (Dahanayake et al. 2018). Although fluorine is the most abundant halogen, evolution has hardly evolved any fluorine containing natural compounds in living organisms apart from mineral bone materials. Therefore, ^19^F is a bioorthogonal NMR reporter in the complex matrix of large biomolecules (Marsh and Suzuki 2014 and Odar et al. 2015). NMR observation of fluorine incorporated into proteins has become increasingly popular in guiding drug discovery and ligand optimization processes throughout the decades after seminal experiments in the 70s (Hull and Sykes 1974, Kitevski-LeBlanc et al. 2012, Arntson and Pomerantz 2016). This evolution was driven by progress in experimental techniques, such as the discovery and use of ^19^F-^19^F through-space scalar couplings (Orton et al. 2021), analysis of ^19^F relaxation (Krempl and Sprangers 2023), ^19^F-paramagnetic relaxation enhancement (PRE) studies (Matei and Gronenborn 2016), or the ^19^F-TROSY methodology (Boeszoermenyi et al. 2019). Consequently, there is an increasing demand for proteins containing fluorine labels, and numerous methods for their preparation have been published so far.

Site-selective incorporation has been achieved using orthogonal *t*RNA/amino acyl synthetase pairs which recognize the fluorinated amino acid building blocks (Galles et al. 2023). Other approaches use auxotrophic host organisms, lacking the biosynthetic pathway to generate the natural amino acid, which can then be replaced by a fluorinated analog (Wang et al. 2003). A third possibility includes the application of certain additives in the growth media of the expression organism, which inhibit defined pathways of the amino acid metabolism, thus reducing the availability of the natural amino acid. An important example for the latter includes the addition of N-(phosphonomethyl)glycine (glyphosate^®^) as an inhibitor of the 5-enolpyruvylshikimate-3-phosphate synthase (EPSPS) blocking the shikimate pathway (Crowley et al. 2012). This enzyme-ligand interaction shuts down the biosynthesis of the aromatic amino acids phenylalanine, tyrosine and tryptophan and facilitates the uptake of fluorinated amino acids or metabolic precursors thereof.

**Fig. 1:**
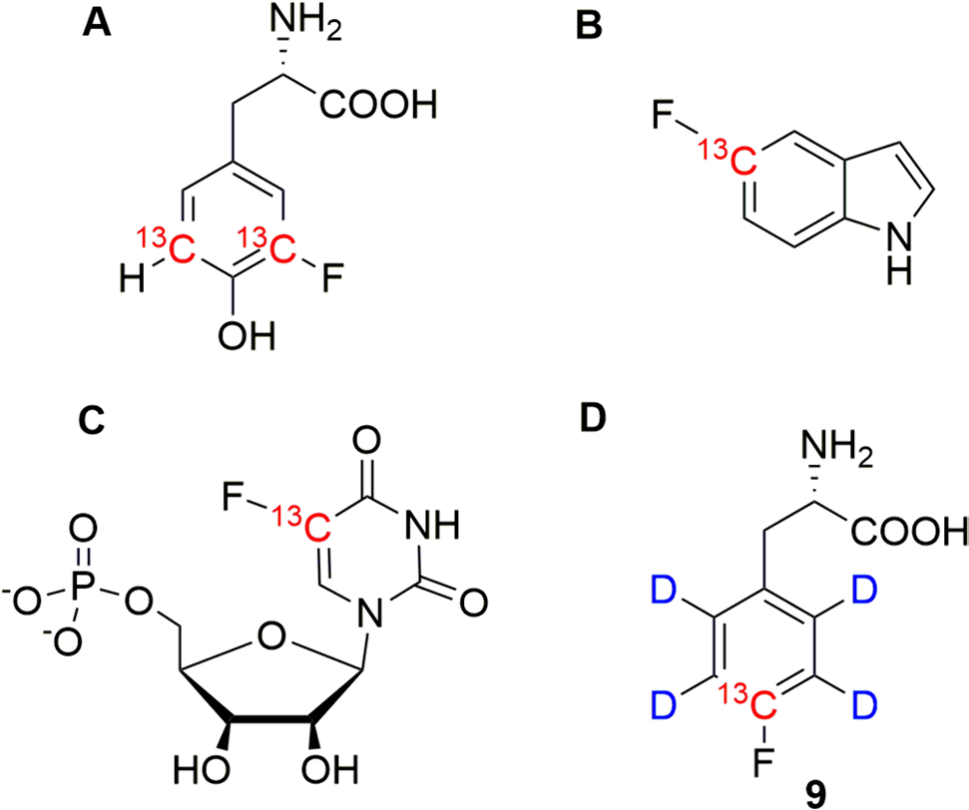
Compounds for introducing ^19^F-^13^C spin systems into biomolecules: [3,5-^13^C_2_]-3-fluorotyrosine for F-Tyr labeling (**A**); [5-^13^C]-5-fluoroindole for F-Trp labeling (**B**); [^19^F-^13^C] fluorouracil (**C**) and the novel [4-^13^C; 2,3,5,6-^2^H_4_]-4-fluorophenylalanine **9** for F-Phe labeling presented in this study (**D**)

Fluorine is considered to be isosteric with hydrogen having a comparable van der Waals radius. Therefore, many reports from literature indicate only minor changes in protein structure upon incorporation of fluorinated amino acids, although the characteristics and functions including stability, folding, dynamic properties and interactions can be significantly altered by fluorine (Welte et al. 2020). Fluorine substituents at aromatic side chains reduce the negative electrostatic potential of the corresponding aryl π-system, thereby changing its nature in contributing to π-π, cation-π, or XH-π interactions (Monkovic et al. 2022). Fluorine-19 is an exceptionally valuable nucleus to study large protein complexes with very high resolution by NMR. In the case of large molecular weight biomolecules, their transverse relaxation is dominated by the dipole–dipole (DD) interaction and chemical shift anisotropy (CSA) mechanisms. Through their (cross-)correlation these two mechanisms contribute to the signal’s different multiplet components with opposite signs. The Transverse Relaxation Optimized Spectroscopy (TROSY) selects for the most favorable component, where DD and CSA mechanism almost cancel out each other, resulting in sharp signals (Pervushin et al. 1997). The TROSY experiment was first introduced for backbone ^15^N-^1^H^N^ nuclei and aromatic ^13^C–H spin systems (Pervushin et al. 1998) and was later transferred to methyl ^13^C side chains (Ollerenshaw et al. 2003; Schütz and Sprangers 2020), taking advantage of dipolar (D–D) cross-correlated relaxation in conceptually similar manner. Recently, the TROSY effect of aromatic carbon ^13^C attached to ^19^F was identified to result in very narrow linewidths even in the case of high molecular weight samples (Boeszoermenyi et al. 2019). Despite of the great potential of the ^19^F-^13^C TROSY technique to characterize structural dynamics and interactions of large biomolecules, corresponding methods for efficiently introducing ^19^F-^13^C spin systems in the target biomolecules are still poorly developed. Notable exceptions contain the application of [3,5-^13^C_2_]-3-fluorotyrosine (Fig. 1**A**; Boeszoermenyi et al. 2019), various ^13^C-fluoroindoles (e.g. [5-^13^C] 5-fluoroindole **B**) as precursors of fluorotryptophan (Maleckis et al. 2021), as well as ^19^F-^13^C fluorouracil **C** to label corresponding RNA segments (Nußbaumer et al. 2020). Here we present a straightforward synthetic procedure to close this methodological gap and expand the toolbox of ^19^F-^13^C spin sources by the F-phenylalanine isotopologue **9** (Fig. 1**D**), which embeds the bioorthogonal ^19^F-^13^C spin system into an otherwise deuterated phenyl ring. This isotope pattern removes two- as well as three-bond ^13^C-^1^H ^2/3^J-couplings leading to narrower signals without the need of proton decoupling during acquisition. As a preliminary first application, we used different concentrations of compound **9** in the *E. coli*-based overexpression of the protein GB1 and determined corresponding incorporation levels.

## Materials and methods

### Synthesis of [4-^13^C, 2,3,5,6-^2^H_4_] 4-fluorophenylalanine 9

**Fig. 2:**
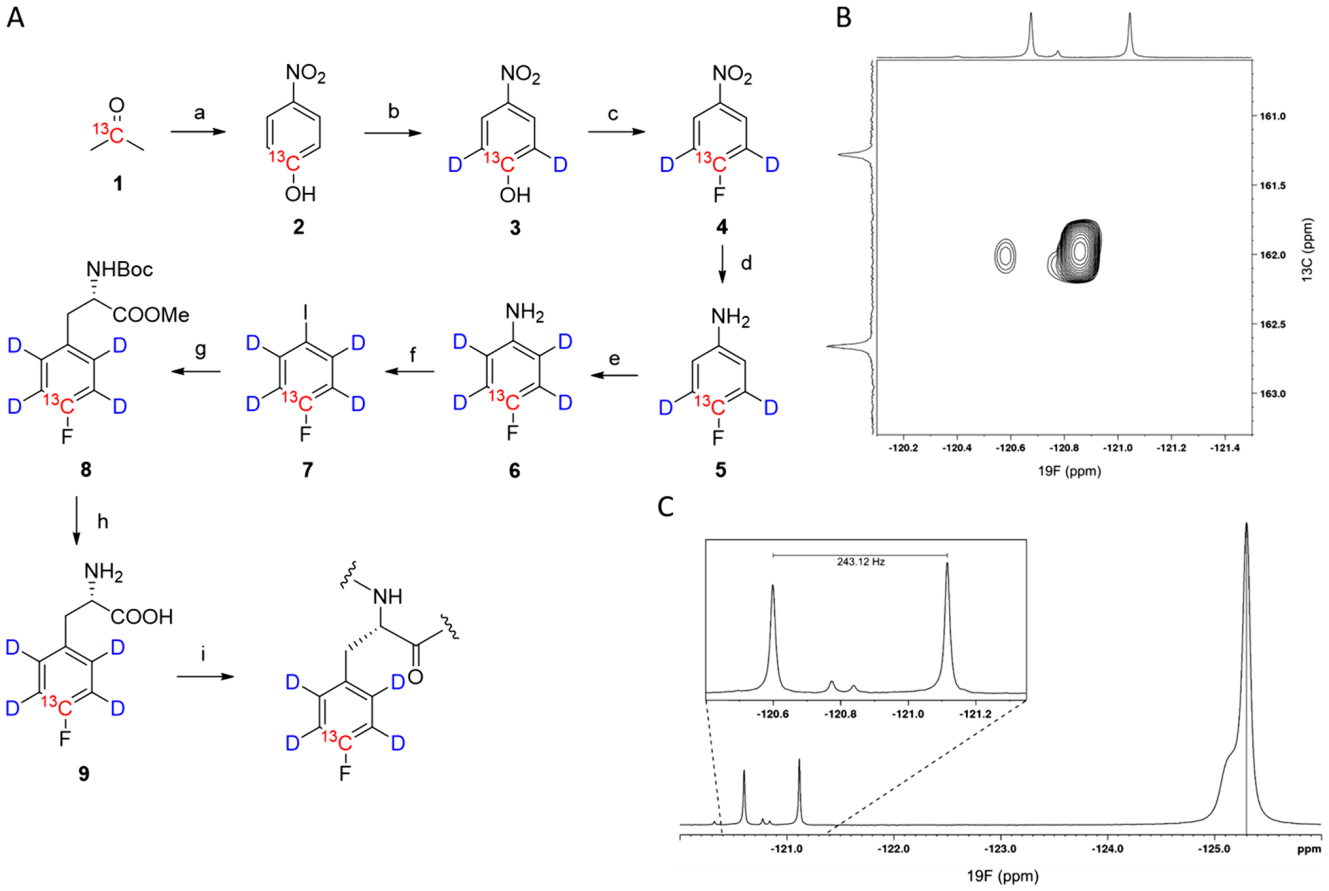
**A** Synthetic route to prepare compound **9** using [2-^13^C] acetone **1** and deuterium oxide as isotope sources. Reagents and reaction conditions: a) aqueous NaOH, nitromalonaldehyde, 7 days, 4 °C, 63%; (b) D_2_O/DCl, microwave irradiation, 170 °C, 60 min., 96%; (c) Phenofluormix®, 110 °C, 24 h, 73%; (d) H_2_, Pd/C 10%, 8 h, 95%; (e) D_2_O/DCl, microwave irradiation, 180 °C, 120 min., 95%; (f) HCl, NaNO_2_, NaI, RT, 30 min. ≥ reflux, 30 min., NaOH, Na_2_S_2_O_3_, 76%; (g) Zn, DMF, N-Boc-3-iodo-l-alanine-methylester, I_2_, Pd_2_dba_3_, SPhos, RT, 24 h, 90%; (h) methanol, LiOH.H_2_O, then dioxane, HCl, 0 °C -> RT, 2 h, 98%; (i) *E coli* overexpression, see main text for details; **B** ^19^F-^13^C HSQC NMR spectrum of compound **9** acquired at 16.4 T. 1D spectra displayed along the shift axes are shown without decoupling. C) 1D ^19^F-NMR of compound **9** acquired at 11.7 T without ^13^C decoupling; KF was added as a chemical shift standard and the corresponding signal referenced to − 125.3 ppm

The synthetic route to access compound **9** is shown in Fig. 2A. [2-^13^C] acetone **1** with an isotopic purity of ~ 99% ^13^C was purchased from CortecNet® (France); N,N′-1,3-bis(2,6-diisopropylphenyl)chloro-imidazolium chloride/CsF (PhenoFluorMix^®^) was prepared according to literature (Fujimoto and Ritter 2015). Sodium nitromalonaldehyde was synthetized from mucobromic acid as described in literature (Fanta 1952). All other reagents were obtained from commercial suppliers and used without further purification. Microwave reactions were performed in an Initiator ^+^ from Biotage^®^. NMR-spectra of compound **9** and synthetic intermediates are shown in the supporting information (SI).

#### [1-^13^C] 4-nitrophenol 2

Compound **2** was prepared by condensation of commercially available [2-^13^C] acetone with nitromalonaldehyde (Lichtenecker 2014). An aqueous NaOH solution (4.4 g in 20 mL) was slowly added to a mixture of sodium nitromalonaldehyde (3.25 g) and [2-^13^C] acetone **1** (1 g) in H_2_O (200 mL) at 0 °C using a dropping funnel. After the addition was complete, the flask was tightly closed and stirred for 6 days at 4 °C. The resulting solution was cooled to 0 °C and 6 N HCl (26 mL) was slowly added. Filtration of the solution resulted in a dark solid, which was taken up in 6 N HCl (26 mL) and boiled gently for 10 min. The warm mixture was filtered, and the two combined filtrates were extracted with diethyl ether (6 × 100 mL). Subsequent drying of the combined organic phases over MgSO_4_ and evaporation of the diethyl ether under reduced pressure yielded a yellow solid. The crude product was purified over a silica gel chromatography column by elution with hexane–ethyl acetate (6:4 v/v). The reaction yielded 1.47 g (63%) of [1-^13^C] 4-nitrophenol **2**. ^1^H NMR (400 MHz, CDCl_3_) δ: 8.19 (dd, J = 9.4 Hz, 2 H, CH_arom__._), 6.92 (dd, J = 9.4 Hz, J = 2.4 Hz, 2 H, CH_arom__._), 5.39 (1H, OH); ^13^C NMR (100.6 MHz, CDCl_3_) δ: 160.94 (^13^CH), 126.16, 115.63 (d, J = 68.0 Hz).

#### [1-^13^C, 2,6-^2^H_2_] 4-nitrophenol 3

A microwave vessel was loaded with [1-^13^C] 4-nitrophenol **2** (338 mg), D_2_O (1.5 mL), and DCl 7 N (0.5 mL). The mixture was irradiated for 1 h at 170 °C. The product was then extracted with diethyl ether (3 × 60 mL). The organic phases were dried over MgSO_4_, and the solvent was evaporated, resulting in 330 mg (96%) of [1-^13^C, 2,6-^2^H_2_]4-nitrophenol **3**, isolated as yellow crystals. ^1^H-NMR spectroscopy analysis indicated quantitative deuterium incorporation at positions 2 and 6. ^1^H NMR (400 MHz, CDCl_3_) δ: 6.92 (d, J = 9.6 Hz). ^13^C NMR (100.6 MHz, CDCl_3_) δ: 160.94 (^13^CH).

#### [4-^13^C, 3,5-^2^H_2_]4-fluoronitrobenzene 4

1.52 g of CsF and 0.68 g of N,N′-1,3-Bis(2,6-diisopropylphenyl)-2-chloro imidazolium chloride were stirred at 140 °C in vacuo for 3 h. The resulting solid mixture was allowed to cool to room temperature (RT) and merged with 142 mg of [1-^13^C, 2,6-^2^H_2_]4-nitrophenol **3** in a round bottomed flask equipped with a reflux condenser under argon atmosphere. Dry toluene (7 mL) was added, and the mixture stirred at 110 °C for 24 h. After completion, the reaction mixture was cooled to RT and filtered through celite, eluting with dichloromethane. The filtrate was then concentrated in vacuo to yield a red oil, which was further purified using a chromatography column with heptane/ethyl acetate (20:1) as the eluent. This step resulted in the isolation of 110 mg (76%) of [4-^13^C, 3,5-^2^H_2_]4-fluoronitrobenzene **4**. ^1^H NMR (400 MHz, CDCl_3_) δ: 8.29 (dd, J_HF_ = 4.5 Hz, J_CH_ = 10.8 Hz).^13^C NMR (100.6 MHz, CDCl_3_) δ: 166.4 (d, J = 257.8 Hz).

#### [4-^13^C, 3,5-^2^H_2_]4-fluoroaniline 5

114 mg (1 mmol) of substrate **4** were dissolved in 4 mL of anhydrous methanol in a Schlenk flask and 40 mg of 10% Pd/C catalyst were added. The flask was purged with argon and then exposed to H_2_ using a hydrogen balloon. The mixture was stirred under H_2_ overnight and then filtered through celite, eluting with dichloromethane. The solvent was removed in vacuo and the resulting residue dissolved in 10 mL of dichloromethane. The dichloromethane solution was washed with 10 mL of 1 M NaOH and 10 mL of brine. The organic phase was dried over MgSO_4_, and the solvent evaporated, resulting in 108 mg (95%) of [4-^13^C, 3,5-^2^H_2_]4-fluoroaniline **5** as a brown oil. ^1^H NMR (400 MHz, CDCl_3_) δ: 6.63 (dd J_CH_ =10.5 Hz, J_HF_ = 4.4 Hz), 3.5 (bs, 2H, NH_2_). ^13^C NMR (100.6 MHz, CDCl_3_) δ: 157.23 (J_CF_ = 257.9 Hz).

#### [4-^13^C, 2,3,5,6-^2^H_4_]4-fluoroaniline 6

In a microwave vessel, 114 mg of [4-^13^C, 3,5-^2^H_2_] 4-fluoroaniline **5** (1 mmol) was mixed with D_2_O (1.5 mL) and DCl 7 N (0.5 mL). The vessel was irradiated for 2 h at 180 °C. After irradiation, 10 mL of 1 M NaOH were added, and the mixture extracted with dichloromethane (3 × 60 mL). The combined organic phases were dried over MgSO_4_ and the solvent was evaporated, yielding 110 mg (95%) of [4-^13^C, 2,3,5,6-^2^H_4_]4-fluoroaniline **6** as a pale brown oil. ^1^H-NMR spectroscopy indicated almost quantitative aryl deuteration with residual protons in position 2 and 3 of < 5%. ^1^H NMR (400 MHz, CDCl_3_) δ: 3.53 (bs, 2H, NH_2_). ^13^C NMR (100.6 MHz, CDCl_3_) δ: 157.23 (J_CF_ = 257.9 Hz).

#### [1-^13^C, 2,3,5,6-^2^H_4_]1-fluoro-4-iodobenzene 7

The procedure was modified from literature (Madden et al. 2019). 116 mg of [4-^13^C, 2,3,5,6-^2^H_4_]4-fluoroaniline **6** (1 mmol) were dissolved in a mixture of 1.3 mL H_2_O and 0.3 mL conc. HCl in a 10 mL round-bottom flask, which was placed in an ice bath at 0 °C. 140 mg of NaNO_2_ in 2 mL of H_2_O were slowly added while maintaining the temperature below 5 °C. After completing the addition, the mixture was stirred for 20 min at 0–5 °C. Next, 332 mg of NaI (2 mmol) in 2 mL of H_2_O were added gradually over 20 min. The mixture was stirred at room temperature for 30 min before heating to reflux for another 30 min. After cooling to room temperature, the solution was neutralized with 1 M NaOH, and then 30 mL of 10% Na_2_S_2_O_3_ solution were added. The aqueous phase was extracted with dichloromethane (3 × 50 mL), and the combined organic layers were dried over MgSO_4_. The solvent was evaporated, yielding a pale-yellow oil, which was further purified by bulb-to-bulb distillation at 90 °C and 20 mbar, resulting in 172 mg (76%) of [1-^13^C, 2,3,5,6-^2^H_2_]1-fluoro-4-iodobenzene **7** as a colorless oil. ^13^C NMR (100.6 MHz, CDCl_3_) δ: 162.84 (d, J_CF_ = 247.0 Hz, ^13^CF).

#### [4-^13^C, 2,3,5,6-^2^H_4_]***N***-[(1,1-dimethylethoxy)carbonyl]-4-fluoro-l-phenylalanine methyl ester 8

Zinc dust (190 mg, 3 mmol) was loaded to a flame dried, argon purged round-bottom flask. Dry DMF (1 mL) was added via syringe followed by a catalytic amount of iodine (40 mg, 0.15 mmol). N-Boc-3-iodo-l-alanine-methylester (329 mg, 1 mmol) was added immediately followed by a catalytic amount of iodine (40 mg, 0.15 mmol), which resulted in a significant rise of temperature. After the solution had come to room temperature again, Pd_2_dba_3_ (22 mg, 0.025 mmol), SPhos (21 mg, 0.05 mmol) and 295 mg of [1-^13^C, 2,3,5,6-^2^H_4_]1-fluoro-4-iodobenzene **7** (1.3 mmol) were added to the flask and left to stir at room temperature for 24 h under argon. The crude reaction mixture was purified using silica gel column chromatography to yield 272 mg (90%) of product **8** as a brown solid. ^1^H NMR (400 MHz, CDCl_3_) δ: 1.42 (9 H, s), 3.01 (dd, 1H, J = 13.9 Hz and 5.6 Hz), 3.11 (dd, 1H, J = 13.9 Hz and 6.0 Hz), 3.72 (s, 3 H), 4.57 (m, 1H), 4.97 (d, 1H, J = 8.4 Hz). ^13^C NMR (100.6 MHz, CDCl_3_) δ: 161.96 (d, J_CF_ = 245.2 Hz, ^13^CF). HRMS (ESI) for C_14_H_16_O_4_^13^CD_4_FN; m/z = 325.1550 ([M + Na]^+^_calc__._ = 325.1553).

#### [4-^13^C, 2,3,5,6-^2^H_4_]4-fluoro-l-phenylalanine 9

ln a 50 mL round bottom flask, 302 mg of [4-^13^C, 2,3,5,6-^2^H_4_]*N*-[(1,1-dimethylethoxy)carbonyl]-4-fluoro-l-phenylalanine methyl ester **8** were dissolved in 7 mL of MeOH, treated with a solution of LiOH·H_2_O (145 mg in 2.5 mL of water) at RT and stirred overnight. Then, the mixture was washed with 2 × 10 mL of diethyl ether. The aqueous layer was acidified with 1 M HCl to pH 2 and extracted with 4 × 15 mL of ethyl acetate. The combined organic phases were washed with brine, dried over MgSO_4_, filtered and concentrated in vacuo. The resulting viscous oil was taken up in 5 mL of dioxane under argon and a mixture of cold 4 M HCl (9 mL) and dioxane (5 mL) was added dropwise over 10 min. at 0 °C. The reaction was stirred for another 10 min at this temperature, then allowed to warm to room temperature and stirred for another 2 h. Dioxane was removed using a rotary evaporator and the resulting solid triturated with 40 mL of diethyl ether and few drops of ethyl acetate. The resulting solid was isolated by filtration to give 220 mg (98%) of product **9**. ^1^H NMR (400 MHz, D_2_O) δ: 3.19 (dd, 1H, J = 14.5 Hz and 7.5 Hz), 3.32 (dd, 1H, J = 14.5 Hz and 5.5 Hz), 4.18 (dd, 1H, J = 7.5 Hz and 5.5 Hz). ^13^C NMR (100.6 MHz, H_2_O) δ: 162.08 (d, J_CF_ = 243.5 Hz, ^13^CF). HRMS (ESI) for C_8_H_7_O_2_^13^CD_4_FN; m/z = 189.1049 ([M + H]^+^_calc__._ = 189.1053).

### Expression and purification of ^19^F/^13^C/^2^H phe GB1

His_6_-TEV-GB1_2−56_ (MKHHHHHHPM SDYDIPTTEN LYFQ/GAMA QYKLILNGC TLKGETTTEA VDAATAEKVF KQYANDNGVD GEWTYDDATK TFTVTE) was expressed in BL21(DE3) phage resistant *E.coli* using a pETM-11 vector system. Cultures were grown in a ^15^N-enriched M9 minimal medium (1 g/L ^15^NH_4_Cl) at 37 °C under agitation (140 rpm) until OD_600_ had reached 0.7. Then 2 g/L glyphosate along with 120 mg/L L-Trp and 120 mg/L L-Tyr and 100/200/400 mg/L (sample 1, 2 and 3) of [4-^13^C, 2,3,5,6-^2^H_4_] 4-fluorophenylalanine **9** were added. After 40 min at 37 °C the temperature was lowered to 28 °C, and after another 15 min cell expression was induced by adding isopropyl-β-D-thiogalactopyranoside (IPTG; 0.4 mM final concentration). The expression culture was incubated for 16 h at 28 °C. Cells were pelleted by centrifugation, resuspended in PBS buffer (137 mM NaCl, 2.7 mM KCl, 10 mM Na_2_HPO_4_, 1.8 mM KH_2_PO_4_, pH 7.4) and lysed by sonication. After centrifugation the supernatant was filtered and purified with a HisTrap FF crude column (GE Life Sciences) using an imidazole gradient (500 mM). The buffer was exchanged to a TEV-cleavage buffer (50 mM Tris-HCl, 0.5 mM EDTA, 1 mM DTT, pH 8) and the protein incubated with TEV protease overnight at 4 °C. The protein was further purified via a reversed HisTrap step. All NMR measurements were performed in a 50mM NaAc buffer (1 µM NaN_3,_ + 1 mM fresh DTT, pH 5.5). Non-^19^F-labeled GB1 (sample 0) was expressed in the same way, without addition of glyphosate, Trp, Tyr and compound **9**. The protein yields for samples 1–3 in 100 mL M9 minimal medium cultures each have been determined to 0.39 mg, 0.32 mg and 0.16 mg, respectively. This corresponds to 500 µl samples with concentrations of 0.12, 0.1 and 0.05 mM.

### NMR experiments

2D ^15^N-^1^H spectra, employing the SOFAST HMQC NMR pulse sequence (*sfhmqcf2gpph*; d1 = 0.2 s, aq time = 0.06 s, 384 points the i.d. (0.1 s), sw of 15.63 ppm (^1^H) × 35 ppm (^15^N), garp ^13^C-decoupling, and, depending on the sample concentration, 4–32 scans) (Schanda et al. 2005), and 1D ^19^F spectra (*zgig*; d1 = 1 s, aq time = 1.0 s, sw of 40 ppm, 8192 scans and waltz16 ^1^H-decoupling scheme) were both acquired at 298 K on a Bruker^®^ Avance Neo 500 spectrometer (11.7 T), using a broadband BBO probe.

1D ^19^F NMR (*zgig*; d1 = 1 s, aq time = 0.5 s, sw of 30 ppm, 1024 scans) and 2D ^13^C -^19^F HSQC (*hsqcetgpsi*, d1 = 1.5 s, aq time = 0.025 s, 128 points in the i.d. (0.012 s), sw 30ppm (^19^F) × 30ppm (^13^C), 128 scans, garp ^13^C-decoupling) spectra were acquired at 298 K on a Bruker® AV III HD + 700 MHz spectrometer (16.4 T) using a quadruple-resonance QCI_F (^1^H, ^13^C, ^15^N, ^19^F) helium cooled cryo-probe. The ^19^F chemical shifts are referenced to KF (δ = −125.3 ppm), which was specifically co-resolved in the sample for referencing. NMR data was processed and analyzed using TopSpin^®^ software.

## Results and discussion

The synthetic procedure to access [4-^13^C_1_, 2,3,5,6-^2^H_4_]-4-fluorophenylalanine **9** (Fig. 2A) started with 2-^13^C acetone **1**, which was converted to ^13^C-nitrophenol **2** by condensation with nitromalonaldehyde as described in earlier work (Lichtenecker 2014). Subsequent deuteration in ortho position was achieved in acidic D_2_O at elevated temperature. Aryl deoxyfluorination was performed using a mixture of N,N-1,3-bis(2,6-diisopropyl-phenyl)chloroimidazolium chloride and CsF (PhenoFluorMix^®^) as described by Fujimoto and Ritter 2015. Reduction to the fluoroaniline **5** creates an electron-rich aryl system where the remaining protons at the aromatic ring can readily be replaced by deuterium (Lichtenecker 2014). Sandmeyer Iodination of compound **5** using NaNO_2_/NaI (Madden et al. 2019) resulted in the formation of a substrate for zinc-mediated Negishi coupling yielding the protected F-phenylalanine **8** (Young et al. 2021).

Deprotection using standard conditions gave the chloride salt of the target labeled amino acid **9**, which was prepared in 8 steps with an overall yield of 27%. The total costs for substrates and reagents needed to synthesize compound **9** can be estimated to a value of < 300€ for 100 mg, given the current average prize for [2-^13^C] acetone of approx. 800€/g from commercial sources. We used compound **9** in the overexpression of a GB1 model protein (see materials and methods for the amino acid sequence), since expression of this protein has been reported in presence of a non ^13^C/^2^H labeled F-Phe previously and allowed us to draw a comparison with this literature data (Boeszoermenyi et al. 2020).

The ^19^F-^13^C HSQC spectrum of compound **9** (Fig. 2B) shows a main resonance of the deuterated aromatic ring system with chemical shifts of 161.97 ppm (^13^C) and − 120.86 ppm (^19^F) and a ^1^J-coupling constant of ^1^J_19F−13C_ = 243.12 Hz (scheme 1 C) and two minor resonances at a ^19^F chemical shift of − 120.6 ppm and − 120.75. Minor resonances originate from non-fully deuterated ortho- and meta-positions of the aromatic ring. ^1^H-NMR, ^19^F-NMR, as well as mass analysis indicate a residual aryl proton content of < 10% (supporting information, figures SI 14, SI 16 and SI 17). In order to quantify incorporation of the new precursor, the BL21(DE3) *E.coli* strain applied was grown in a minimal medium containing ^15^N-ammonium chloride, glyphosate, tyrosine, tryptophan and different concentrations of compound **9** (100 mg/L = sample 1, 200 mg/L = sample 2 and 400 mg/L = sample 3).

**Fig. 3:**
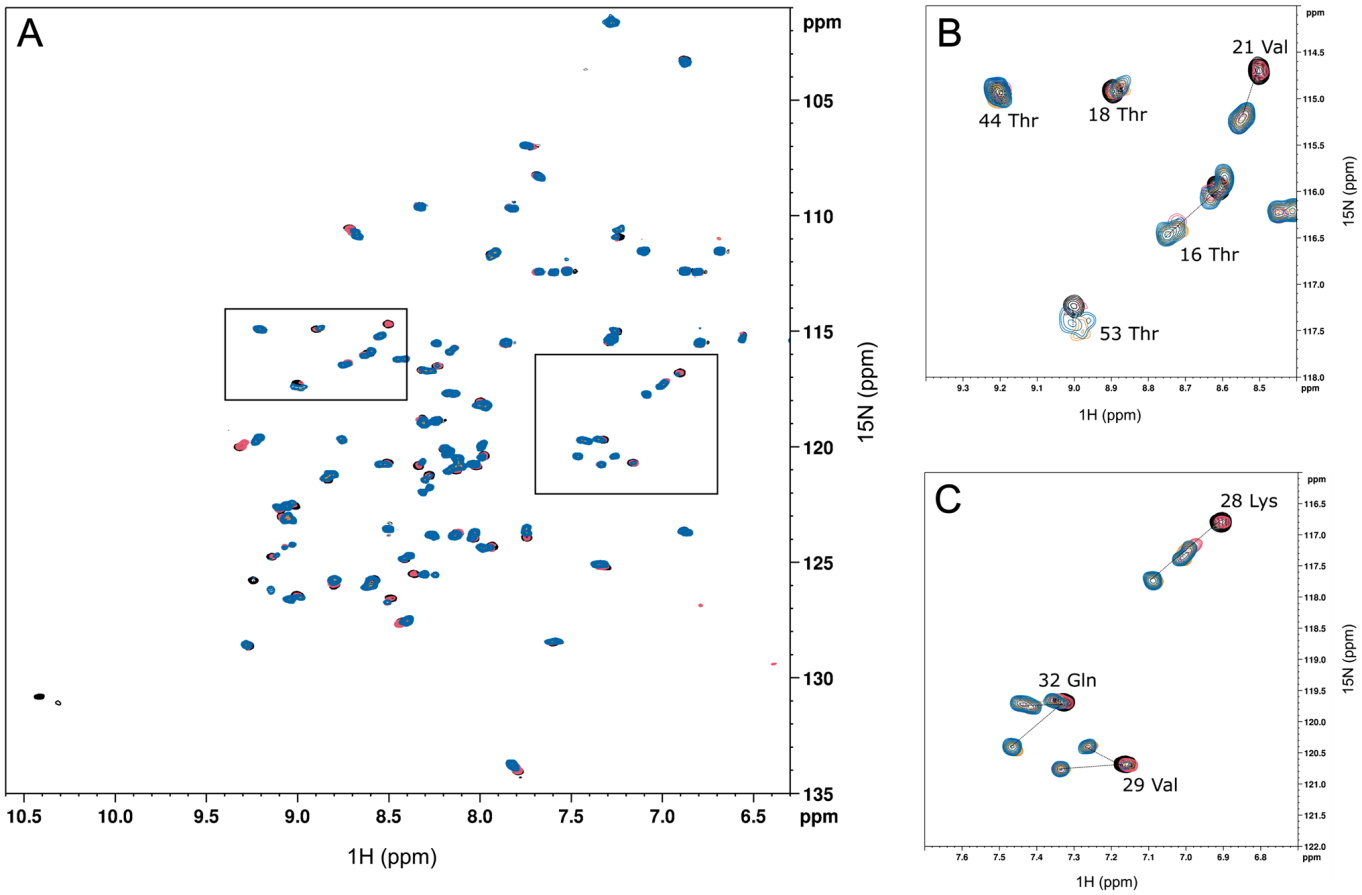
Full ^1^H-^15^N-HMQC NMR spectra of samples 0–3 (black, pink, orange, blue) acquired at 11.7 T (**A**); including expansions of the ^1^H-^15^N-HMQC NMR spectra featuring the residues 44Thr, 18Thr, 16Thr, 53Thr and 21Val (**B**) and 28Lys, 32Gln and 29Val (**C**). Shifted resonances belonging to the same residues are connected with a dashed line

The target protein was expressed as a TEV protease cleavable His6 fusion protein using HisTrap column purification. An additional sample of GB1 was prepared in absence of these additives except ^15^N ammonium chloride (sample 0). We compared backbone ^1^H-^15^N spectra of GB1 with and without ^19^F/^13^C/^2^H side chain labeling (Fig. 3; the individual spectra are shown in the supporting information; figure SI 18-SI 20), which indicated chemical shift changes of signals, especially in the helical region (Asp22-Gly38). For some resonances, even two new peaks appeared (e.g., Val29 and Gln32, Fig. 3C), which supports the assumption of two conformational states being in a thermodynamic equilibrium, as observed in ^19^F-NMR spectra. We utilized this chemical shift change to determine the level of ^19^F/^13^C/^2^H-Phe incorporation by comparing resonance intensities in the 2D ^1^H-^15^N spectra of GB1 with and without Phe side-chain labeling for peaks that do not overlap and for which the assignment of the shifted peaks is unambiguous. The signals resulting from Ala20, Val21, Lys28, Val29, Gln32, and Asn37 were thus analyzed and incorporation levels of 55% (sample 1), 74% (sample 2) and 79% (sample 3) were determined in this way.

**Fig. 4:**
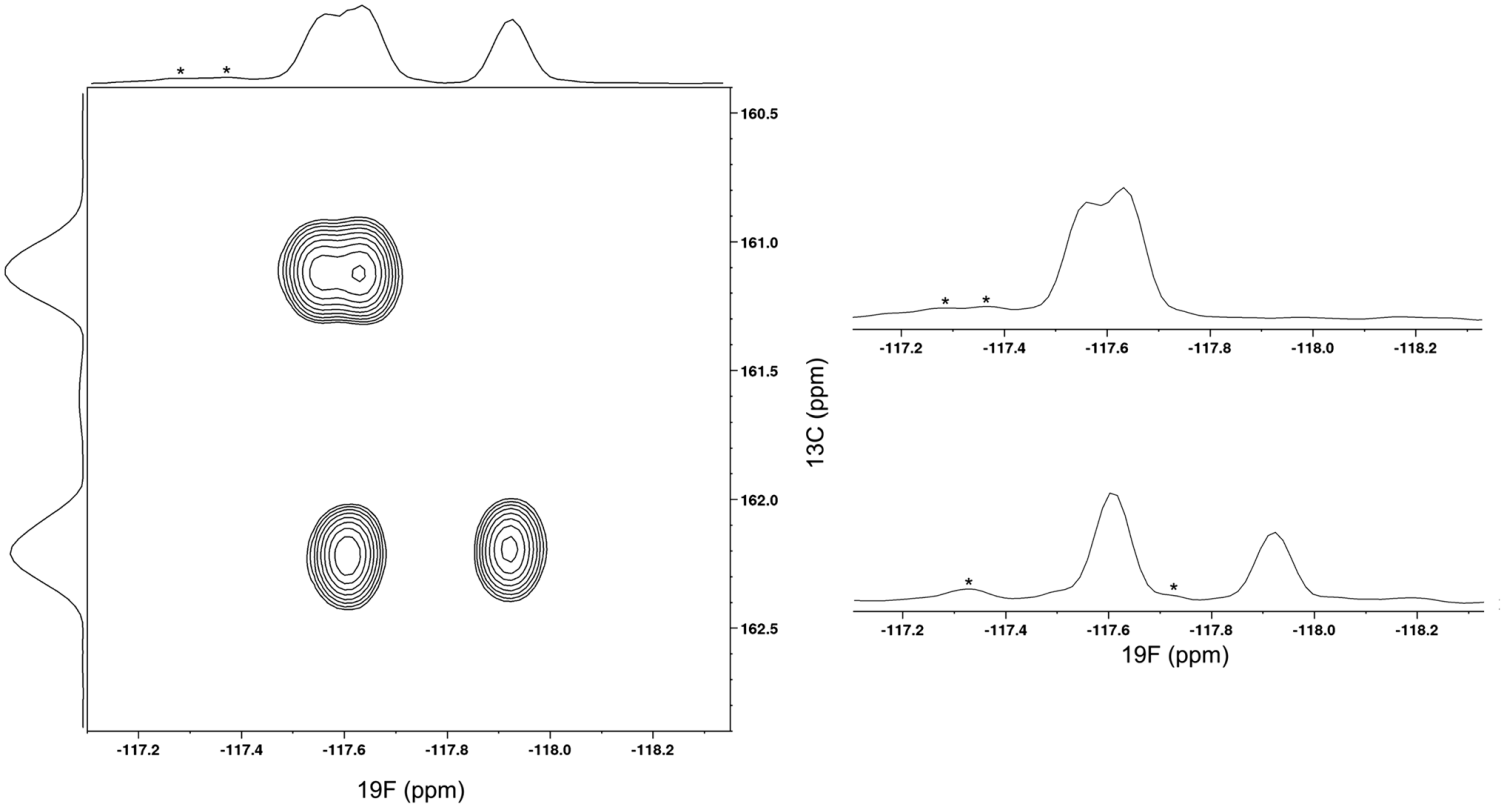
^19^F-^13^C HSQC NMR spectrum of the ^19^F-Phe GB1 (sample 2) acquired at 16.4 T, including sum projections and 1D slices taken from ^13^C chemical shifts of 161.1 ppm (top right) and 162.2 ppm (bottom right). Asterisks mark the minor signals originating from non-fully deuterated meta-positions of the aromatic ring

The GB1 sequence features two phenylalanine residues (Phe30 and Phe52). Figure 4 shows the ^19^F-^13^C correlation of a HSQC spectrum of GB1 (sample 2), indicating both being replaced by the ^19^F/^13^C/^2^H aryl system. However, this spectrum shows four signals, which indicates the presence of GB1 species featuring one F-Phe due to non-quantitative incorporation of compound **9**. This interpretation is substantiated by the corresponding ^19^F-^13^C HSQC of sample 3 (supporting information; figure SI 21), which shows an intensity decrease in two of the four signals with increasing incorporation levels. This observation suggests, that at a higher content of labeled F-Phe the difluorinated GB1 species dominates. The splitting of signals into multiple resonances may as well, at least partially, result from differently populated conformers in an equilibrium on a slow exchange NMR time scale, which cannot be fully excluded at this stage. A similar explanation was hypothesized in earlier work by Boeszoermenyi et al. (2020).

However, in the ^19^F-NMR of fluorinated GB1 reported in this publication, only one F-Phe signal is split, whereas in our case the signal at 161.1 ppm in the ^13^C frequency displays this effect as well (Fig. 4). This additional finding is a result of enhanced resolution, partly attributed to the deuteration pattern in the fluorinated side chain. In accordance with the literature, the three tyrosines of GB1 were not affected and not replaced by any fluorinated species (Boeszoermenyi et al. 2020). Despite the wide chemical shift range of fluorine, signals of the same kind of ^19^F containing side chains at different residue positions usually cluster in a narrow ppm window. Figure 4 reveals the importance of the additional ^13^C dimension to fully resolve these resonances.

## Conclusion

Methods like ^19^F-^13^C TROSY are effective in reducing linewidth and enhancing resolution in large protein complexes. While these methodological advantages have been proven to be widely applicable e.g., in the case of the 42 kDa maltose binding protein (Boeszoermenyi et al. 2019) or the 180 kDa proteasome 20 S alpha 7 subunit (Boeszoermenyi et al. 2020), a significant need for customized labeling strategies still remains. To address this need, we have introduced a robust synthetic route to access a ^2^H/^13^C F-phenylalanine isotopologue from low-cost isotope sources. The deuteration pattern we applied also has the advantage of thus not needing ^1^H decoupling in ^19^F-^13^C NMR, which usually requires extra, specialized hardware. In the first application of this compound, we overexpressed the protein GB1 using a standard BL21(DE3) *E. coli* system, which resulted in high incorporation levels at phenylalanine residues without observing any replacement of tyrosine. With the development of the triple labeled phenylalanine **9**, we provide the protein NMR community with an additional tool to further explore the ^19^F-^13^C TROSY effect on high molecular proteins for future applications. We envision the possibility of synchronistic NMR observations of differently labeled complex biomolecules, both in vitro and even in living cells with fluorine acting as the central bio-orthogonal reporter nucleus within a complex biological matrix. Furthermore, mammalian cell cultures have demonstrated high tolerability towards fluorinated phenylalanine (Westhead and Boyer 1961) and fluorinated aromatic amino acids have been incorporated into proteins using human cell lines, recently (Costantino et al. 2024). An effective, high yielding synthesis of heavy isotope containing F-Phe isotopologues provides the perspective to transfer the labeling method from *E. coli* to other protein expression systems such as insect-cell, yeast, or mammalian cells in the near future.

## Supplementary Information

Below is the link to the electronic supplementary material.

Supplementary material 1 (PDF 2661.3 kb)
