# Supplementary material for: Decorating phenylalanine side-chains with triple labeled 13C/19F/2H isotope patterns
Source: J Biomol NMR. 2024 Mar 21;78(3):139–47. doi: 10.1007/s10858-024-00440-z (PMC11491416; doi:10.1007/s10858-024-00440-z)
Supplement: Supplementary file 1 — Supplementary material 1 (PDF 2661.3 kb) [file 10858_2024_440_MOESM1_ESM.pdf]

Decorating phenylalanine side-chains with triple labeled $^{13}C/^{19}F/^{2}H$ isotope patterns

Giorgia Toscano,[a,c] Julian Holzinger,[b] Benjamin Nagl,[d] Georg Kontaxis,[e] Hanspeter Kählig,[d] Robert Konrat[b] and Roman J. Lichtenecker*[a,f]

[a] G. Toscano, R.J. Lichtenecker
Christian Doppler Laboratory for High-Content Structural Biology and Biotechnology, Institute of Organic Chemistry, University of Vienna, Währingerstr. 38, 1090 Vienna, Austria.
E-mail: roman.lichtenecker@univie.ac.at

[b] J. Holzinger, R. Konrat
Christian Doppler Laboratory for High-Content Structural Biology and Biotechnology, Department of Structural and Computational Biology, Max Perutz Labs, University of Vienna, Campus Vienna Biocenter 5, 1030 Vienna, Austria.

[c] G. Toscano
University of Vienna, Vienna Doctoral School in Chemistry (DoSChem), Währinger Str. 42, 1090 Vienna, Austria.

[d] B. Nagl, H. Kählig
Institute of Organic Chemistry, University of Vienna, Währinger Str. 38, 1090 Vienna, Austria.

[e] G. Kontaxis
Max Perutz Laboratories, Department of Structural and Computational Biology, Campus Vienna Biocenter 5, 1030 Vienna, Austria.

[f] R.J. Lichtenecker
MAG-LAB, Karl-Farkas-Gasse 22, 1030 Vienna, Austria.

Supporting information

Table of contents

Page

1. NMR of isotope labeled compounds……………………………………………2
   - ^{1}H NMR Compound **2**……………………………………………2
   - ^{13}C NMR Compound **2**……………………………………………2
   - ^{1}H NMR Compound **3**……………………………………………3
   - ^{13}C NMR Compound **3**……………………………………………3
   - ^{1}H NMR Compound **4**……………………………………………4
   - ^{13}C NMR Compound **4**……………………………………………4
   - ^{1}H NMR Compound **5**……………………………………………5
   - ^{13}C NMR Compound **5**……………………………………………5
   - ^{1}H NMR Compound **6**……………………………………………6
   - ^{13}C NMR Compound **6**……………………………………………6
   - ^{13}C NMR Compound **7**……………………………………………7
   - ^{1}H NMR Compound **8**……………………………………………7
   - ^{13}C NMR Compound **8**……………………………………………8
   - ^{1}H NMR Compound **9**……………………………………………8
   - ^{13}C NMR Compound **9**……………………………………………9
   - ^{19}F NMR Compound **9**……………………………………………9
2. Mass spectroscopy of compound **9**……………………………………………10
3. ^{1}H-^{15}N-HMQC NMR spectra of samples 0 – 3……………………………………………11
4. ^{19}F-^{13}C-HSQC NMR spectrum of sample 3……………………………………………14

1. NMR of isotope labeled compounds

^{1}H NMR Compound **2**

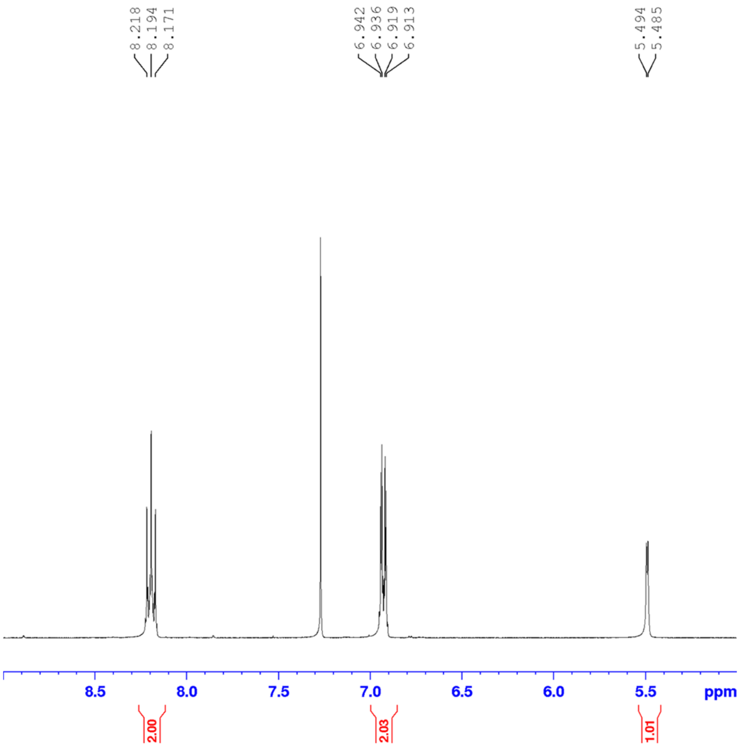

Figure SI 1

^{13}C NMR Compound **2**

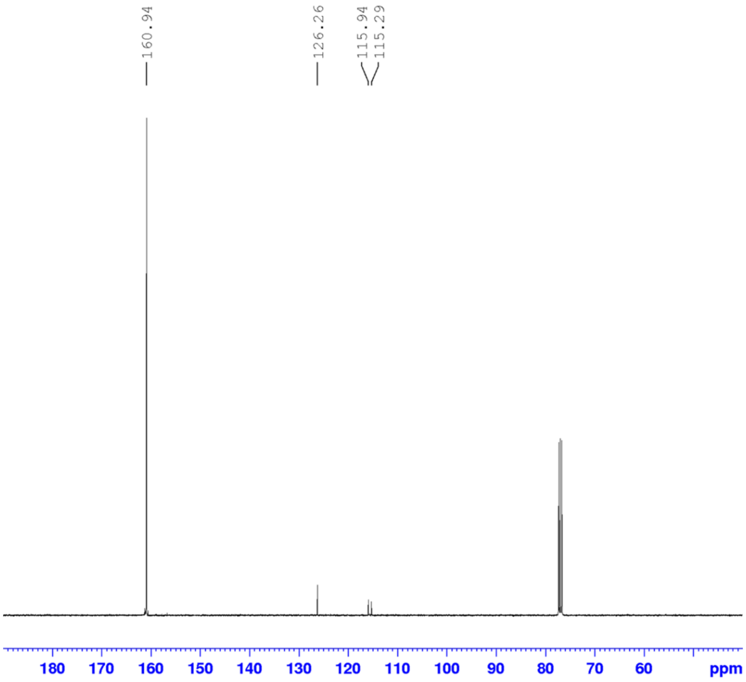

Figure SI 2

^{1}H NMR Compound **3**

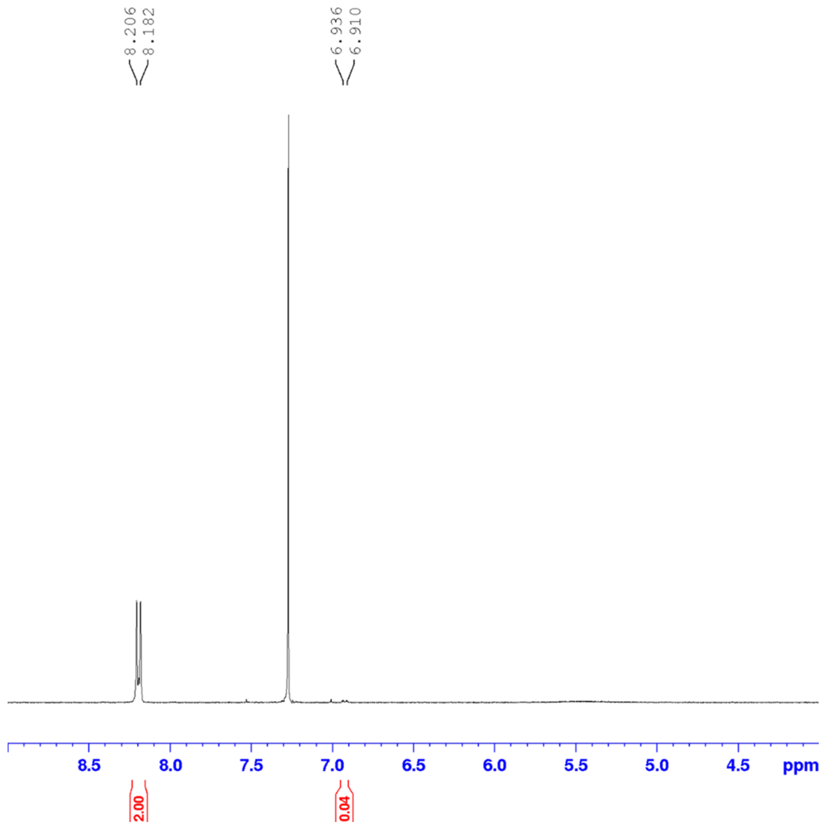

Figure SI 3

^{13}C NMR Compound **3**

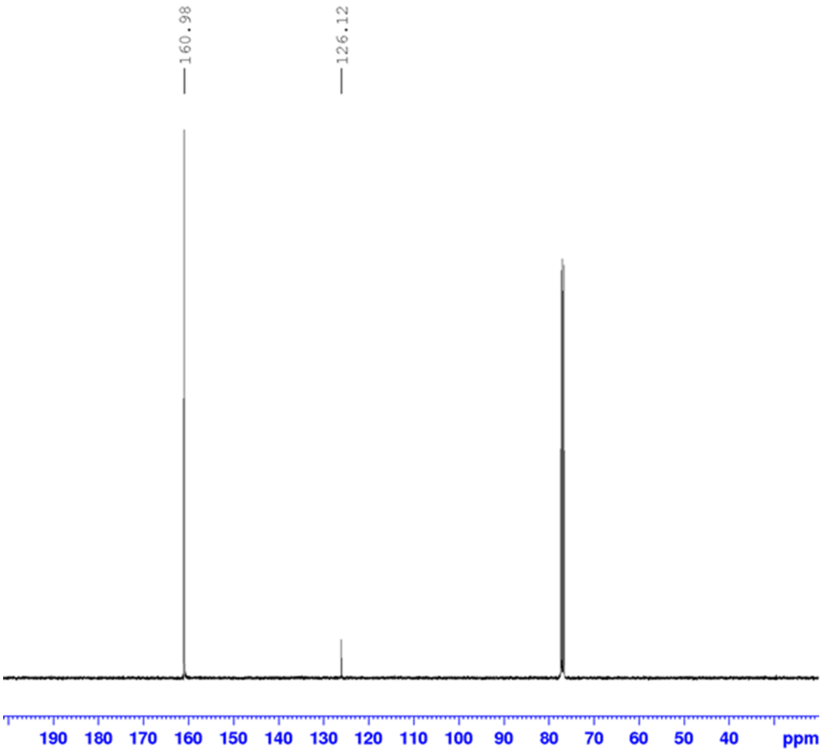

Figure SI 4

^{1}H NMR Compound **4**

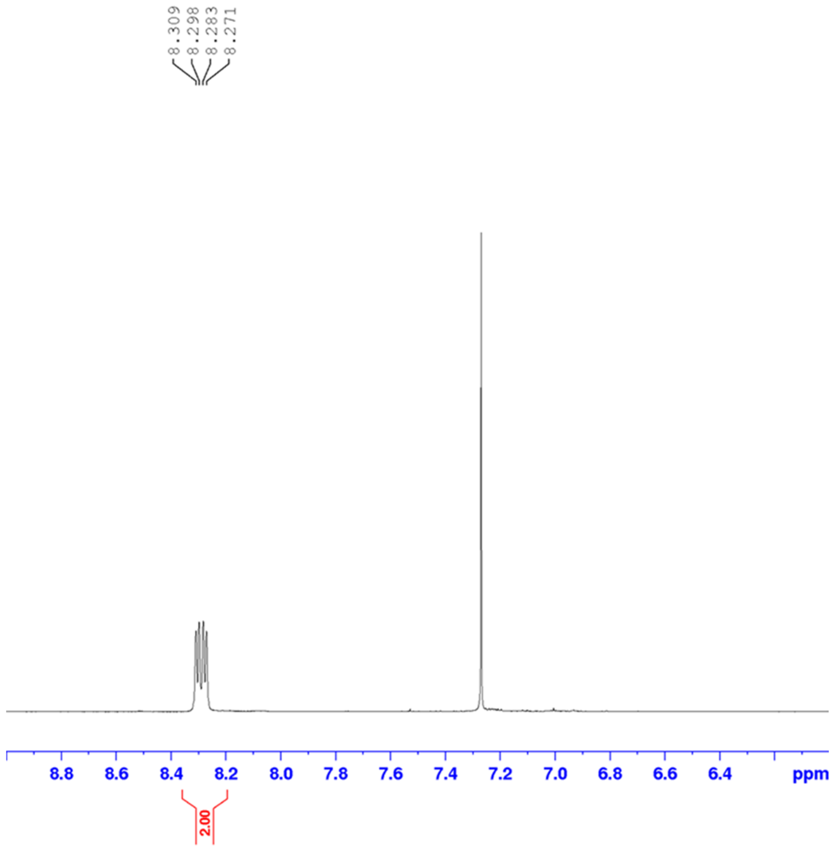

Figure SI 5

^{13}C NMR Compound **4**

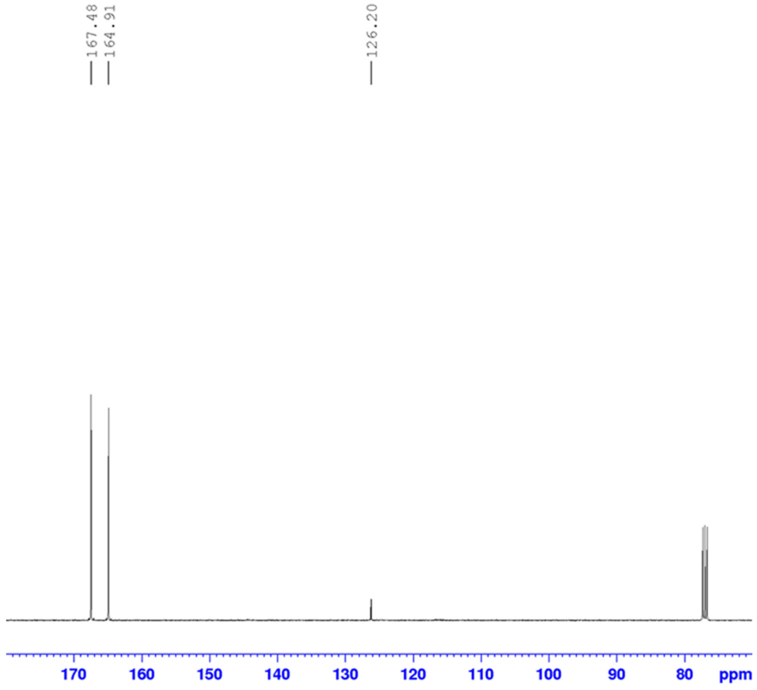

Figure SI 6

^{1}H NMR Compound **5**

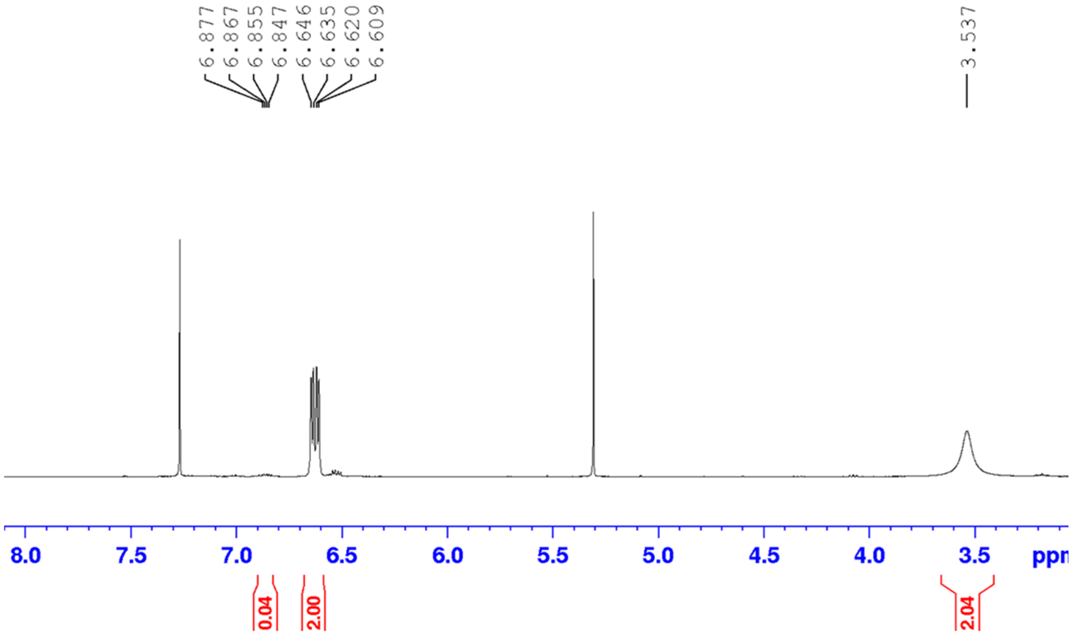

Figure SI 7

^{13}C NMR Compound **5**

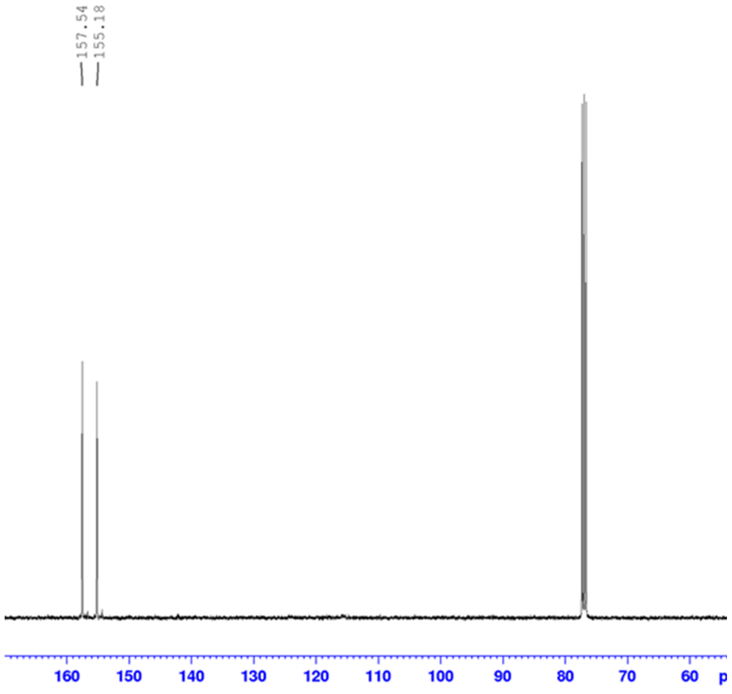

Figure SI 8

^1H NMR Compound **6**

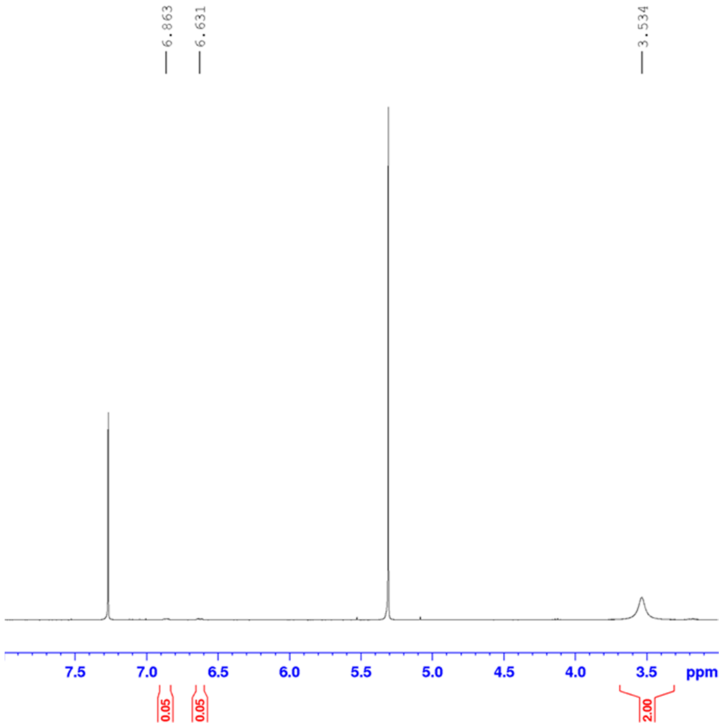

Figure SI 9

^{13}C NMR Compound **6**

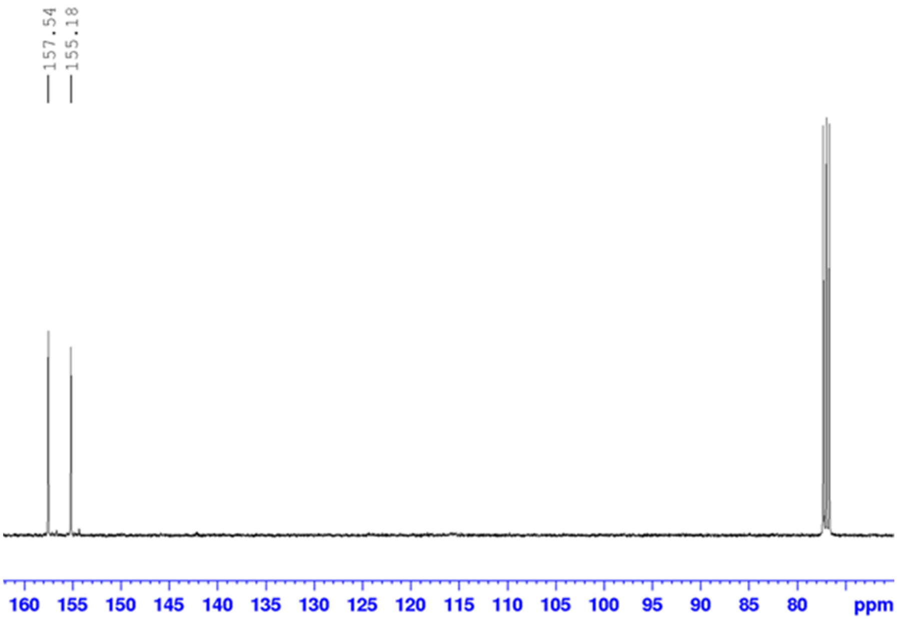

Figure SI 10

^{13}C NMR Compound **7**

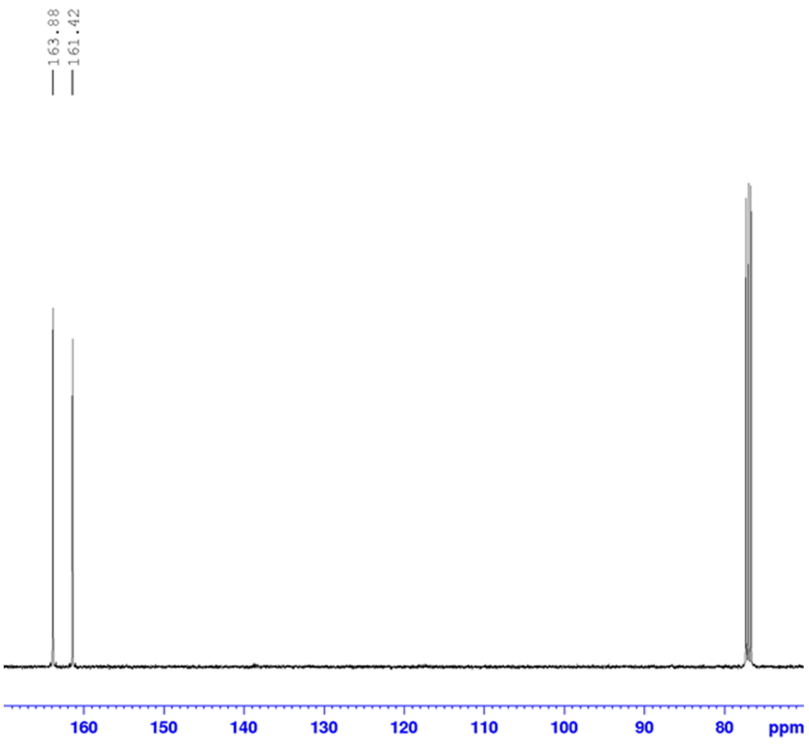

Figure SI 11

^{1}H NMR Compound **8**

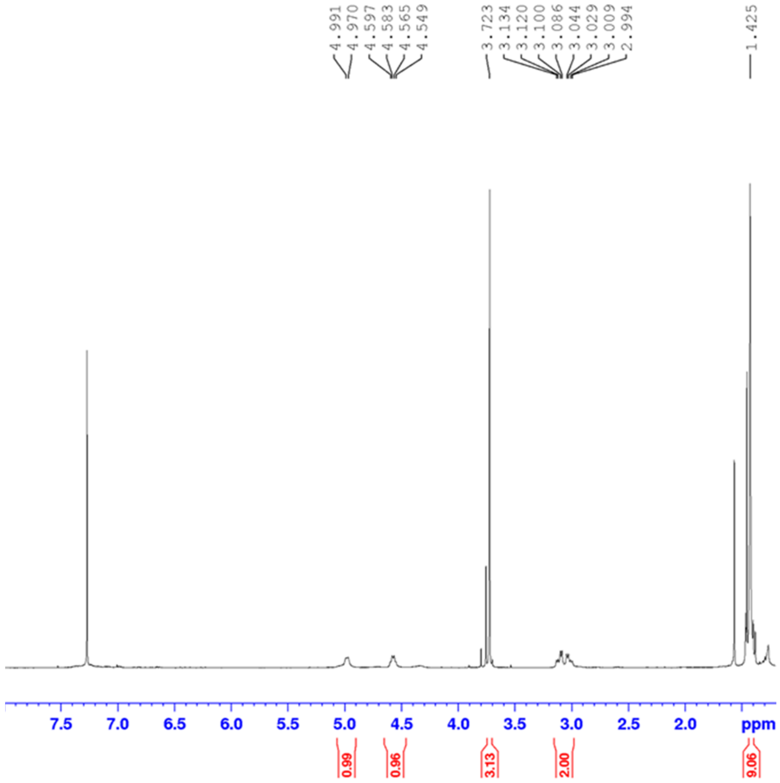

Figure SI 12

^{13}C NMR Compound **8**

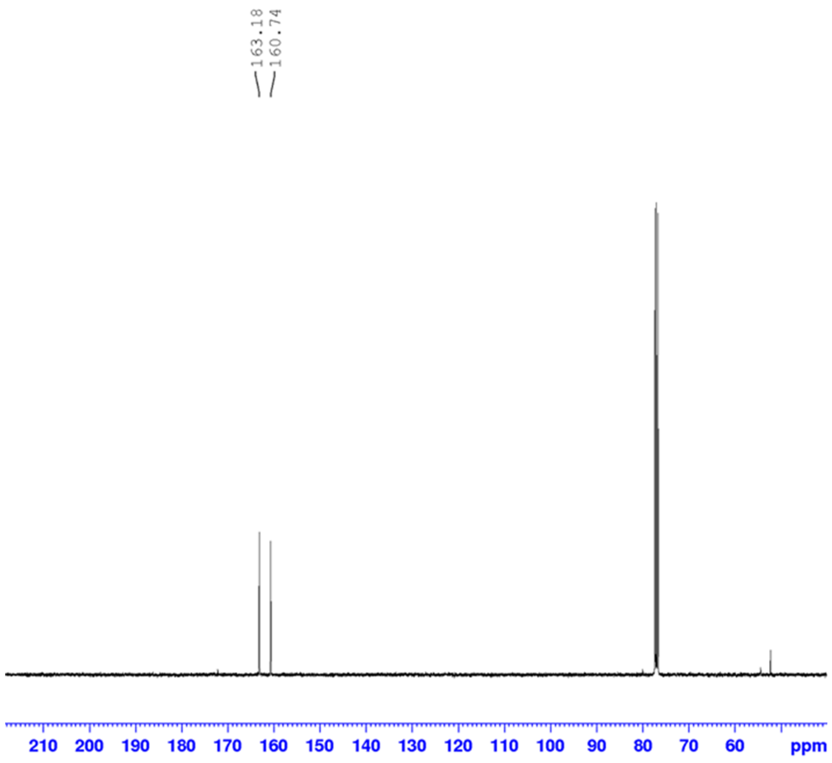

Figure SI 13

^{1}H NMR Compound **9**

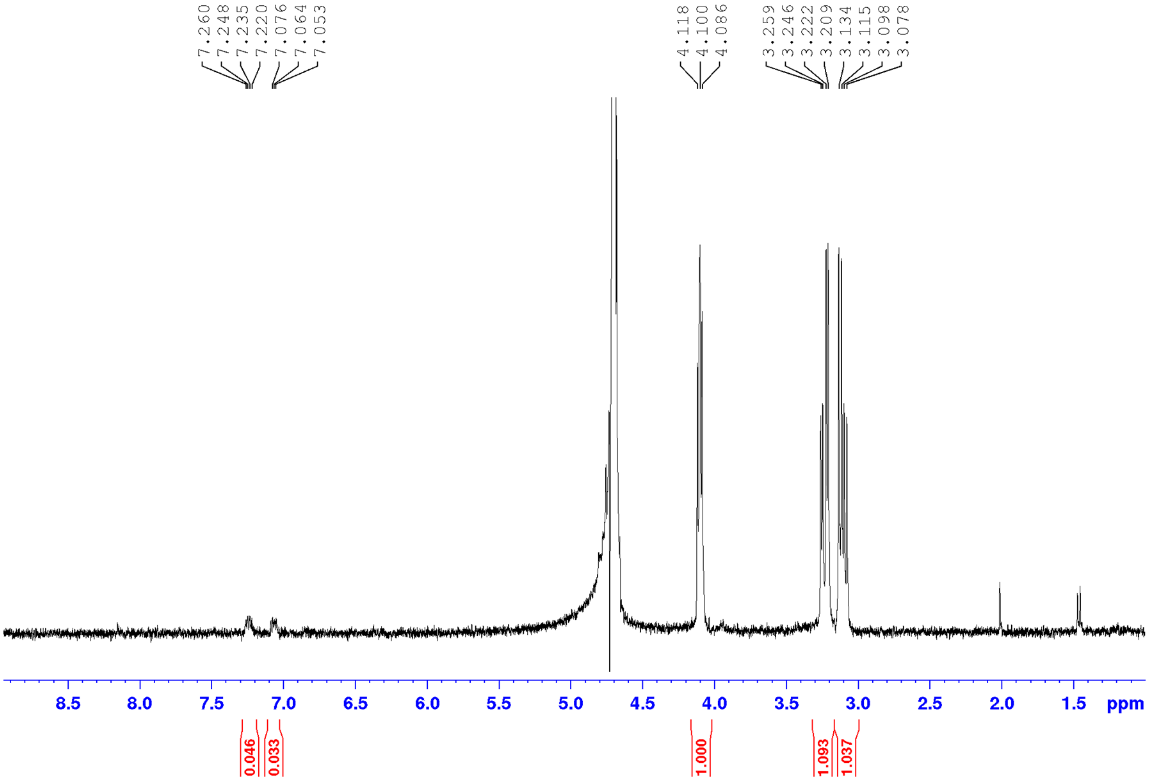

Figure SI 14

^{13}C NMR Compound **9**

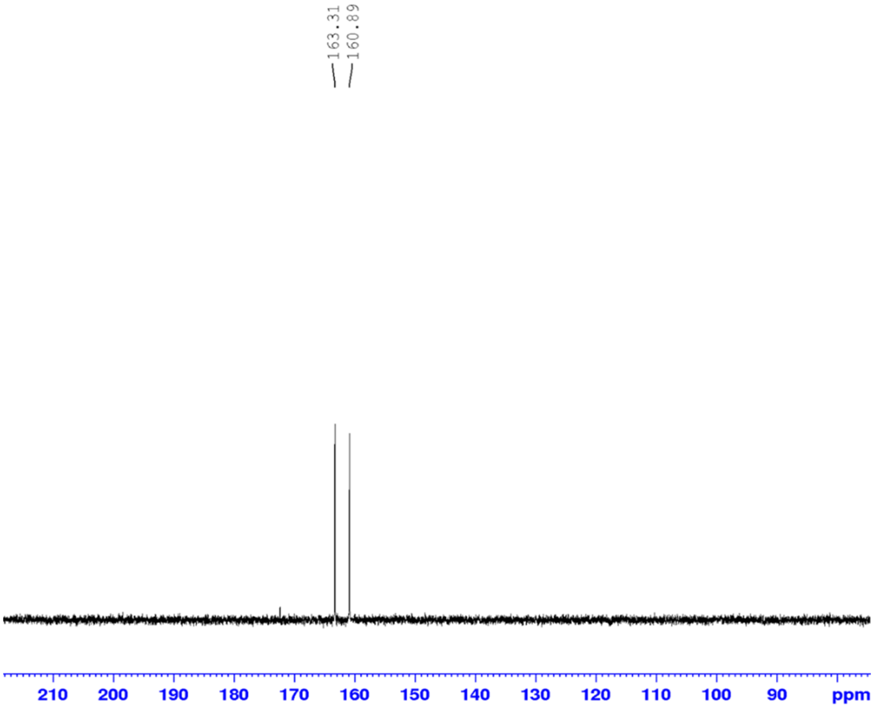

Figure SI 15

^{19}F NMR Compound **9**

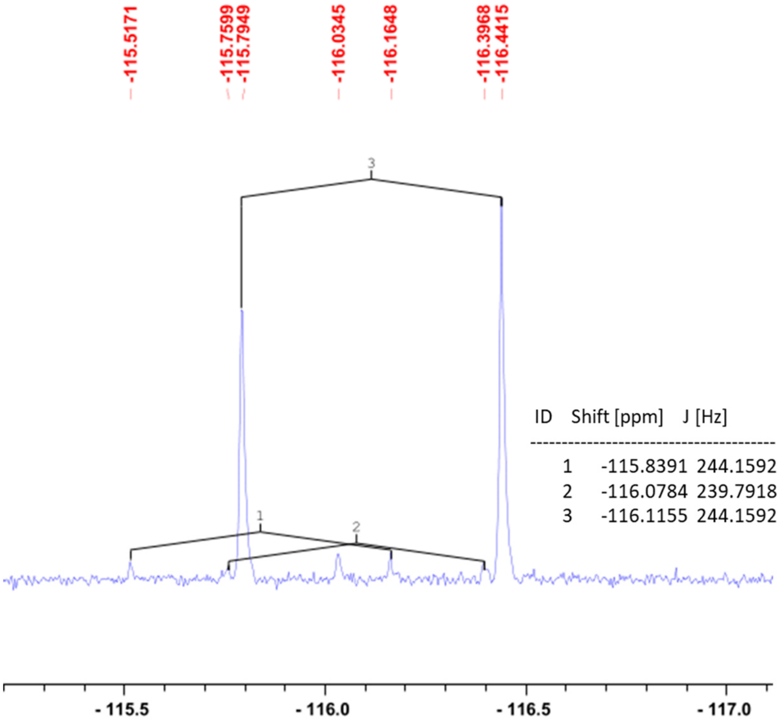

Figure SI 16: ^{19}F NMR of compound **9** (expansion). The major doublet (3) can be assigned to compound **9** fully deuterated at the aromatic positions. Minor doublets (1) and (2) result from compound **9** isotopologues with partial aryl protonation. The minor signal at -116.03 shows no ^{13}C coupling and is most likely derived from a compound **9** isotopologue featuring ^{12}C-^{19}F due to residual carbon-12 (~1%) in the acetone starting compound of the synthetic route.

2. Mass spectroscopy of compound 9

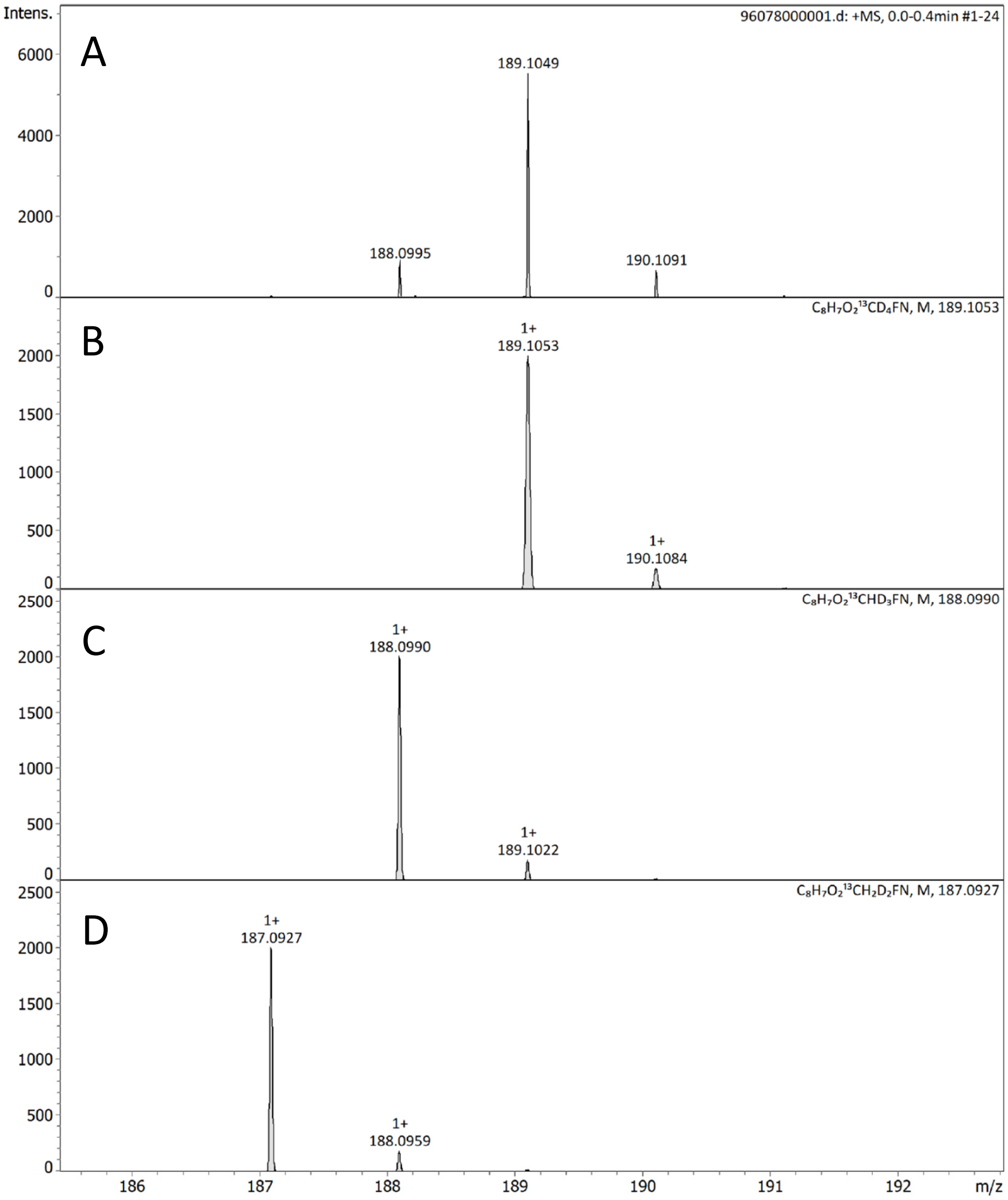

Figure SI 17: MS-Spectra of compound **9** showing the experimental data (A) and calculated masses of fully aryl-deuterated compound **9** (B), a compound **9** isotopologue with one aryl proton (C) and a compound **9** isotopologue with two aryl protons (D). The spectrum was recorded on a MaXis UHR-TOF-MS from Bruker® from a sample in acetonitrile/MeOH + 1% H_2O.

3. ^{1}H-^{15}N-HMQC NMR spectra of samples 0 – 3

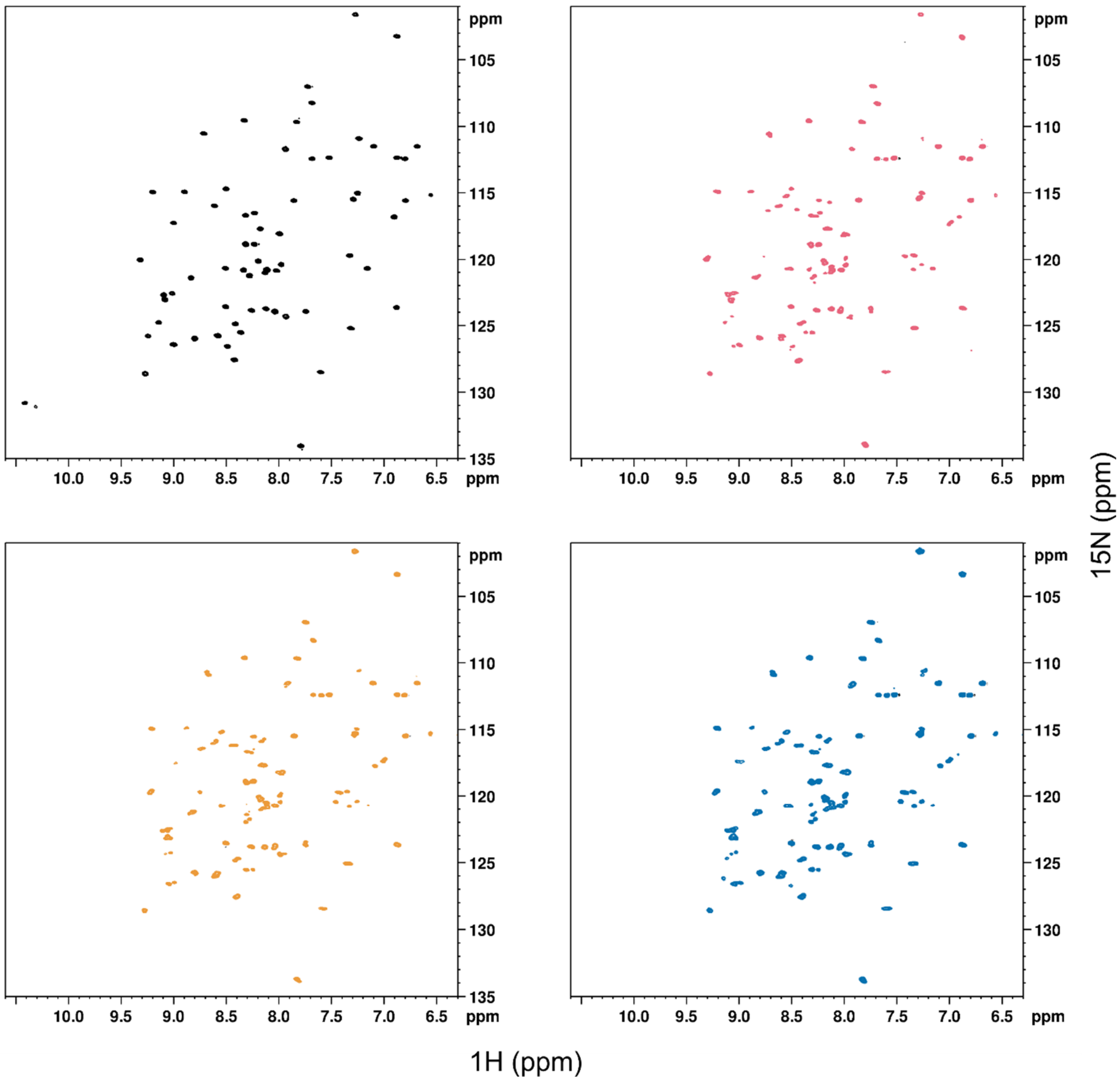

Figure SI 18: Full ^{1}H-^{15}N-HMQC NMR spectra of samples 0 – 3 (black, pink, orange, blue) acquired at 11.7 T (compare Fig.3 (A) in the main manuscript).

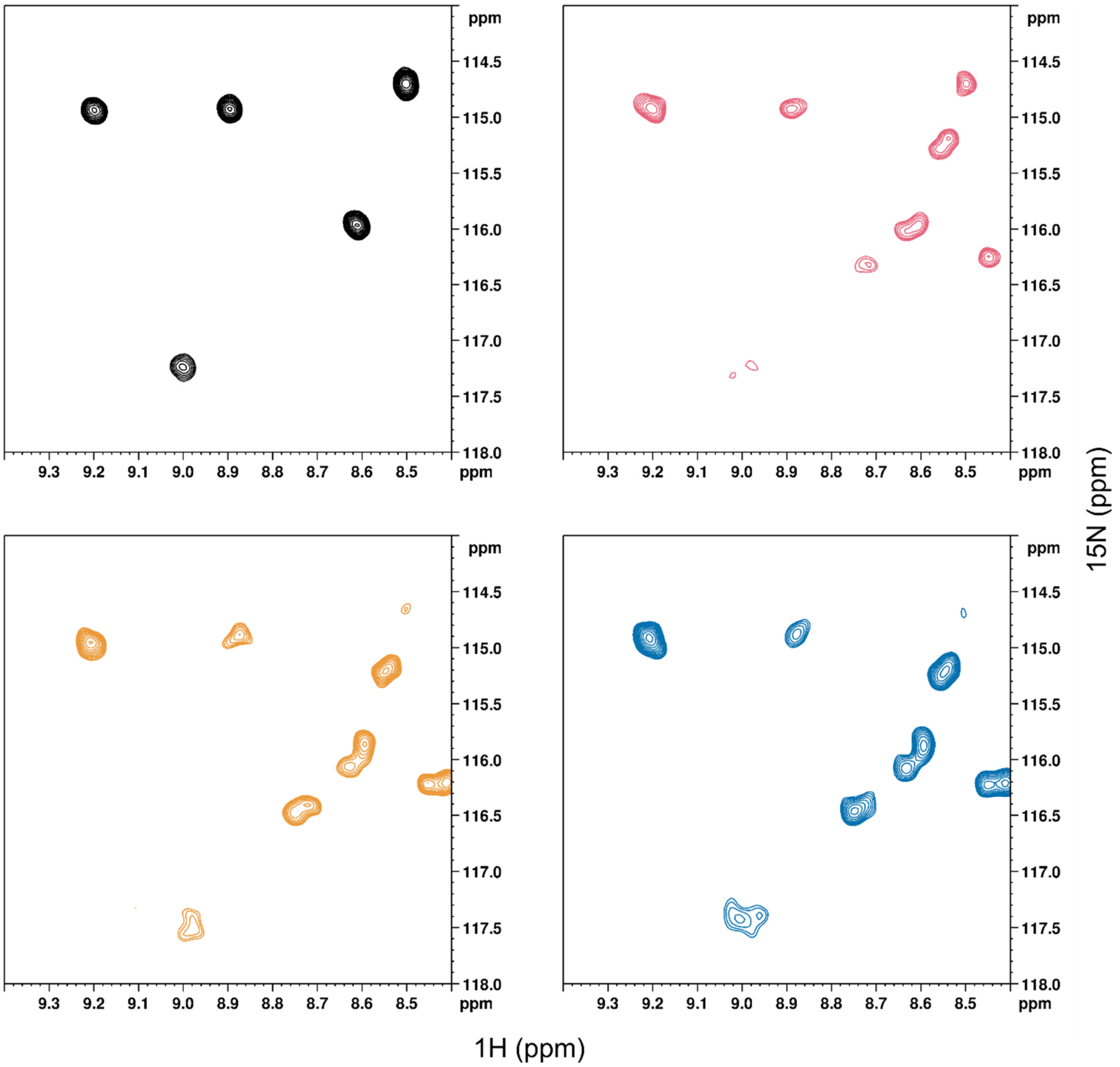

Figure SI 19: Expansion of the ^{1}H-^{15}N-HMQC NMR spectra of samples 0 – 3 (black, pink, orange, blue) acquired at 11.7 T (compare Fig.3 (B) in the main manuscript).

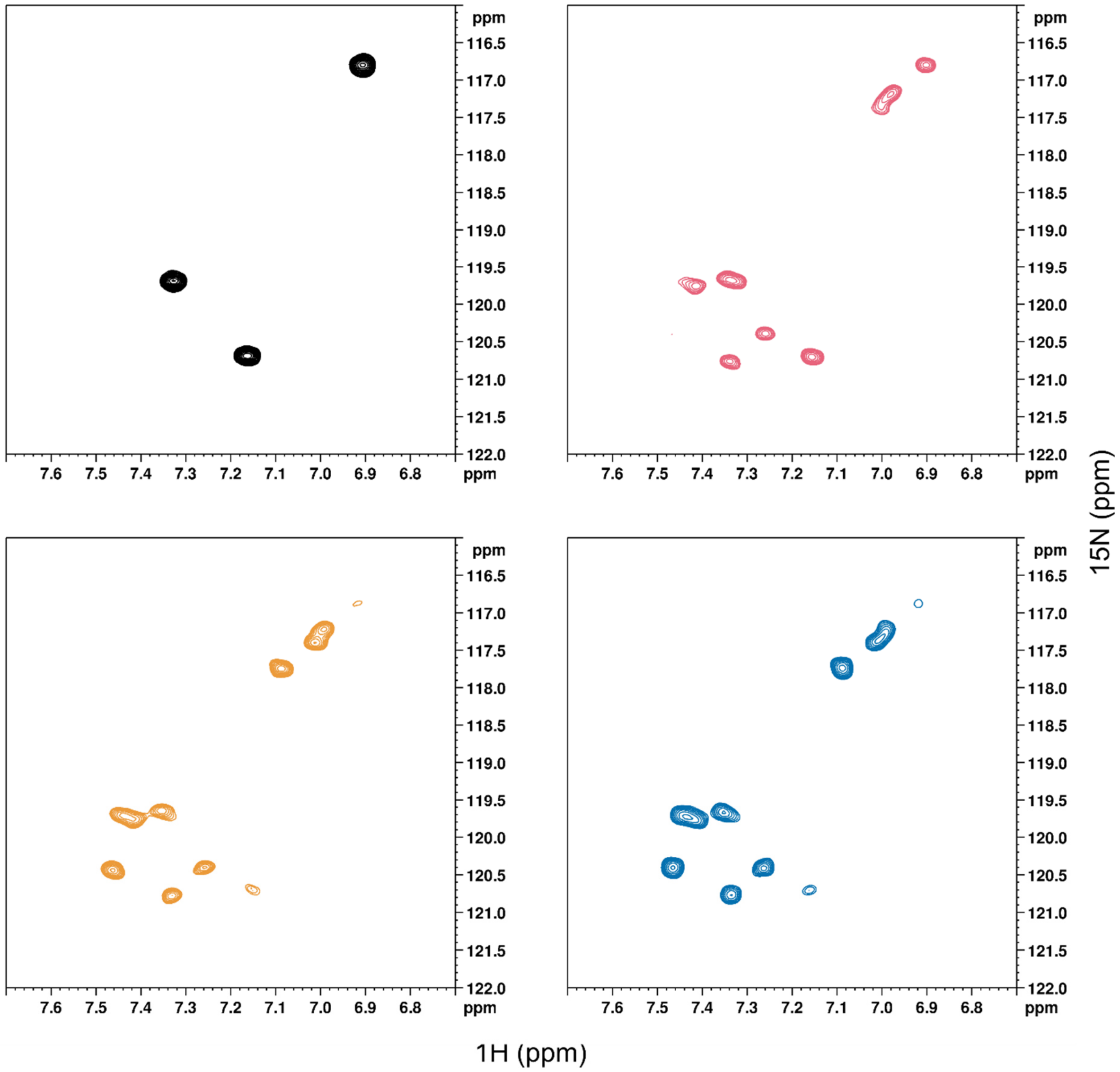

Figure SI 20: Expansion of the ^{1}H-^{15}N-HMQC NMR spectra of samples 0 – 3 (black, pink, orange, blue) acquired at 11.7 T (compare Fig.3 (C) in the main manuscript).

4. ^{19}F-^{13}C-HSQC NMR spectrum of sample 3

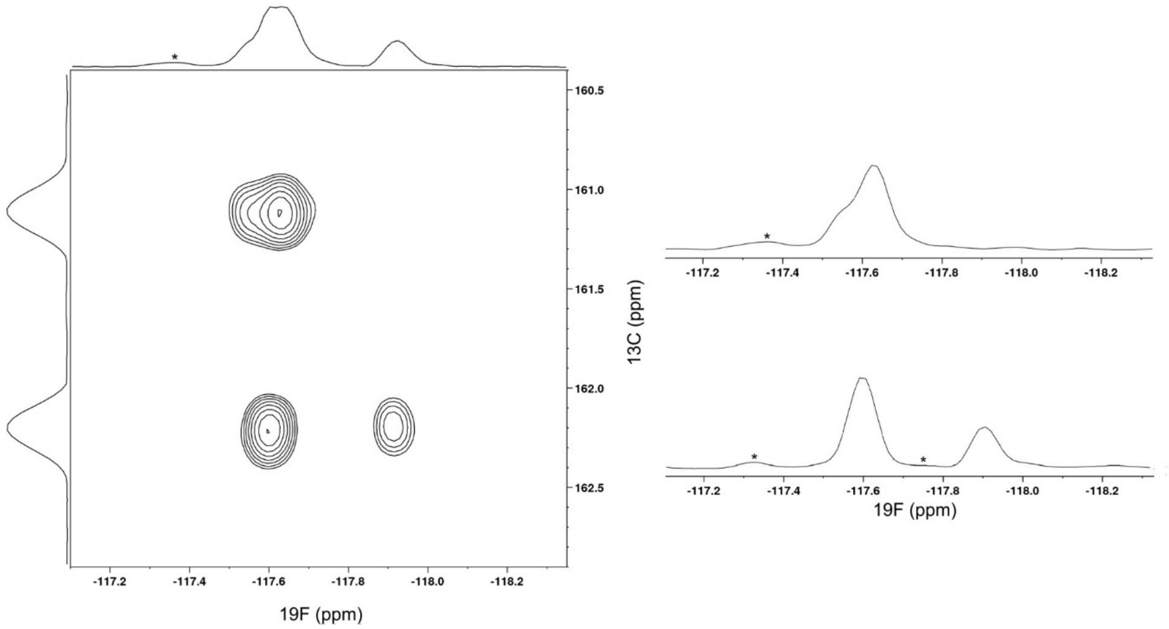

Figure SI 21: ^{19}F-^{13}C HSQC NMR spectrum of the ^{19}F-Phe GB1 (sample 3) acquired at 16.4 T, including sum projections and 1D slices taken from ^{13}C chemical shifts of 161.1 ppm (top right) and 162.2 ppm (bottom right). Asterisks mark the minor signals originating from non-fully deuterated meta-positions of the aromatic ring.
